# Supplementary material for: Experimental demonstration of magnetic tunnel junction-based computational random-access memory
Source: Npj Unconv Comput. 2024 Jul 25;1(1):3. doi: 10.1038/s44335-024-00003-3 (PMC11287819; doi:10.1038/s44335-024-00003-3)
Supplement: Supplementary file 1 — Supplementary Information [file 44335_2024_3_MOESM1_ESM.pdf]

Supplementary Information

Experimental demonstration of magnetic tunnel junction-based computational random-access memory

Yang Lv[1], Brandon R. Zink[1], Robert P. Bloom[1], Hüsrev Cılasun[1], Pravin Khanal[2], Salonik Resch[1], Zamshed Chowdhury[1], Ali Habiboglu[2], Weigang Wang[2], Sachin S. Sapatnekar[1], Ulya Karpuzcu[1] and Jian-Ping Wang[1*]

[1]Department of Electrical and Computer Engineering, University of Minnesota, Minneapolis, Minnesota 55455, USA

[2]Department of Physics, University of Arizona, Tucson, Arizona 85721, USA

[*]e-mail: jpwang@umn.edu;

Supplementary Note S1

CRAM demonstration hardware. The simplified circuit diagram is shown in Fig. S1. It describes the essential part of the CRAM demonstration hardware involving the MTJs and CRAM architecture. The conventional parts, such as digital interface of DAC and ADC, gate controls of transistors, and various power supply and reference voltages, are not shown in the simplified circuit diagram. As shown in the diagram, each MTJ is connected to a transistor and a DAC output channel on both of its terminals, respectively. The DAC output channels generate voltages needed for CRAM operations. The transistors are connected to a common line labeled as 'LL,' short for logic line. The LL is also connected via another transistor, T0, to a pseudo ground, which is implemented by a transimpedance amplifier reference to ground. The output of the transimpedance amplifier is fed into an ADC. Overall, this design is intended to emphasize the demonstration of CRAM logic operations, which is primarily realized by the circuitry on the left side of LL. It is equivalent to a 2T1M CRAM in its logic mode. The part on the right side of LL of this design is a simplified variant of sensing amplifier which trades memory read bandwidth for simplicity of the circuit.

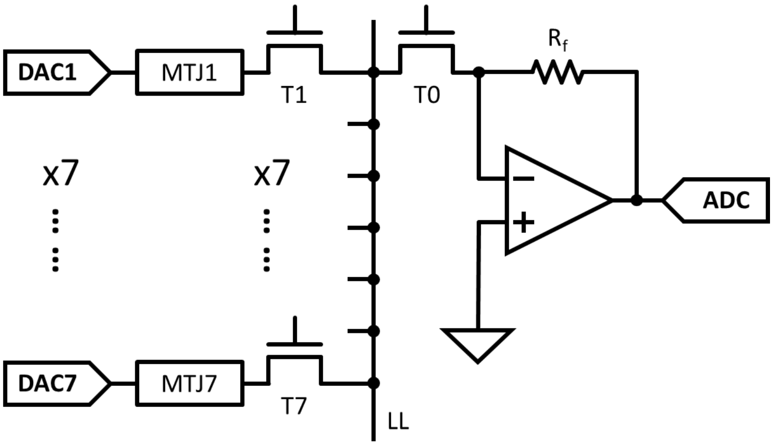

Supplementary Figure S1. Simplified circuit diagram of the CRAM demonstration hardware. The essence of the hardware is equivalent to a 1×7 CRAM with a modified architecture to emphasize logic operation. The memory write (and logic) operation can be performed by turning T0 on (off), updating relevant voltages among DAC1 through DAC7, and momentarily turning on corresponding transistors among T1 through T7. Note that other supporting components and circuits for the experiment, such as the MCU, power management, and connectors and switches, on the main board and the connection board are not shown in this diagram for simplicity.

Specifically, to execute a CRAM logic operation, T0 is first turned off to leave the LL floating. Then appropriate voltages are updated on relevant DAC channels among any of DAC1 to DAC7 corresponding to MTJ1 to MTJ7. Lastly, transistors corresponding to the involved cells (for both input and output cells for the logic operation) are turned on momentarily to establish a VCL circuit equivalent for a certain pulse width. In this way, the intended logic operation is executed. Note that although arbitrary voltages can be applied on involved MTJs, in this work, the output cell is always given 0 V (grounded), while all input cells are given the same voltage, V_{logic}.

To execute a CRAM memory write operation, T0 is turned on to ground LL. Then the following steps of updating DAC voltages and turning on corresponding transistors of the involved cells are the same as those for CRAM logic operation. The only difference is whether the LL is grounded or left floating. Since LL is grounded, the involved cells are applied voltages specified by their corresponding DAC channels.

To execute a CRAM memory read operation, a small bias voltage is updated to the DAC channel corresponding to the cell to be read. Note that only one cell can be read each time. Then both the

corresponding transistor to the target MTJ and T0 are turned on. Therefore, the read bias voltage is applied on the target MTJ, and a current is flowing through the pseudo ground and is sensed by the transimpedance amplifier. Lastly, the output voltage of the transimpedance amplifier is sampled by the ADC. Based on the R_f of the transimpedance amplifier and the read bias voltage, the resistance of the MTJ in the target cell can be obtained.

Supplementary Note S2

MTJ properties. The MTJs used in this work are the perpendicular low-RA CoFeB-MgO ones with stack structure of Si/SiO$_2$/Ta(3)/Ru(6)/Ta(4)/Mo(1.2)/Co$_{20}$Fe$_{60}$B$_{20}$(1)/MgO(0.9)/Co$_{20}$Fe$_{60}$B$_{20}$(1.4)/Mo(1.9)/Ta(5)/Ru(7) with numbers in brackets indicate thickness of the layer in nm. The MTJs are fabricated into circular shapes with size of 100 nm in diameter. The resistance vs. voltage results of the seven MTJs used in this study are shown in Fig. S2. These MTJs exhibit TMR ratios of approximately 100%, RA product of about 18 Ω μm^2, and they are switched under about ±350 mV voltage bias.

The fabricated MTJ array shows yield of approximately 81%. MTJs that do not show TMR signal and electrical (STT) switching are considered 'unacceptable.' Yield is defined as the ratio of MTJs observed to be 'acceptable,' as opposite to 'unacceptable.'

As described in the main text Methods section, after dicing the fabricated array are dived, mounted, and wire-bonded, external bias magnetic fields are applied to each of the seven MTJs. Fig. S2(a) shows an MTJ's typical electrical switching characteristics under various perpendicular bias magnetic field. External magnetic field favors either the P or AP state making that state to be switched to by electrical voltage or current more easily. For example, increasing the external bias magnetic field would favor AP state and increase or decrease the absolute value of voltage required to switch from AP to P state or P to AP state, respectively. In the CRAM demonstration experiment, each of seven MTJs in the experiment has an independent mechanical mechanism to manipulate a permanent magnetic to provide individually different bias magnetic field to each of the MTJs. Then resistance-voltage measurements are run continuously and repeatedly while the results are displayed by the software in real-time. In this way, appropriate tuning of each bias magnetic field can be achieved so that the resistance-voltage curves of all seven MTJs are fairly symmetrical on the voltage axis and are as close to each other as possible. Fig. S2(b) show the collection of seven MTJs after such tuning process that are used in the CRAM experiments, include the memory operation, logic operations, and the full adder demonstrations.

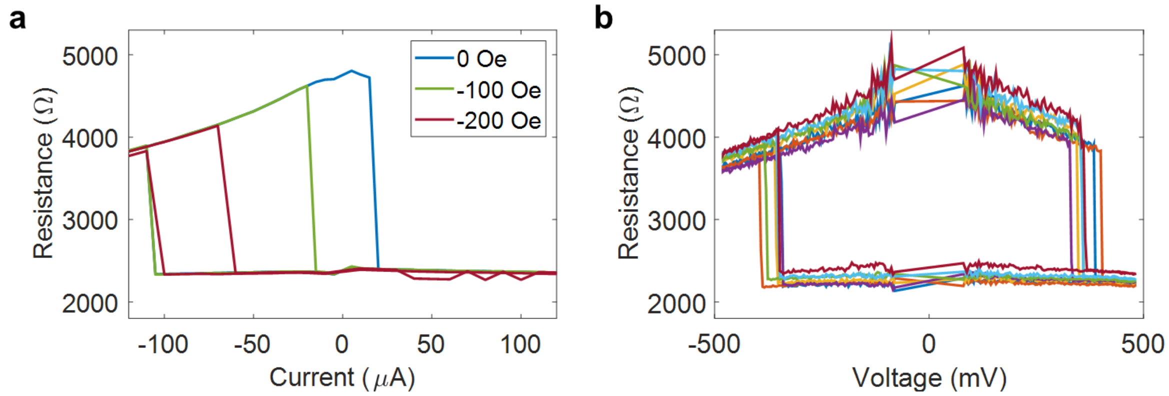

Supplementary Figure S2. Electrical switching of MTJ. a MTJ resistance vs. current under various external perpendicular magnetic fields. The result shows how external magnetic field affects the critical switching current or voltage for AP-to-P and P-to-AP transitions. **b** MTJ resistance vs. voltage of a set of seven MTJs used in the CRAM experiments. The data points with bias voltage under 80 mV are omitted due to large noise. These results were taken after adjusting bias magnetic fields so that the electrical behavior of all seven MTJs are as close to each other as possible.

Supplementary Note S3

CRAM memory write operations. After the DC voltage sweep data are collected for each of the seven MTJs, CRAM memory write operations are tested on these MTJs with various write pulse amplitudes and width. Each MTJ is written with '0' and '1' alternatively for a large number of repeats with certain write pulse parameters (amplitude and width). The cell state is read and recorded after each write operation. Based on the intended write data stream and the actual readout of the cell state data stream, the write success rate and the probability of switching can be calculated. Then similar measurements are repeated with different pulse parameters. These studies help determine an appropriate set of pulse parameters for CRAM write operations with satisfactory memory write error rate. Figure S3 shows the switching probability vs. write pulse amplitude with various pulse widths of one of the seven MTJs. The switching probability curve shows the expected sigmoidal-like shape. And the voltage required for switching decreases with pulse width. In the experiments, the optimal memory write pulse parameters are identified for the MTJs to ensure a low write error rate. And then the average write error rate is verified to be less than 1.5×10^{-4} by a memory test of 10,000 repeats (n = 10000) with the configured write pulse parameters.

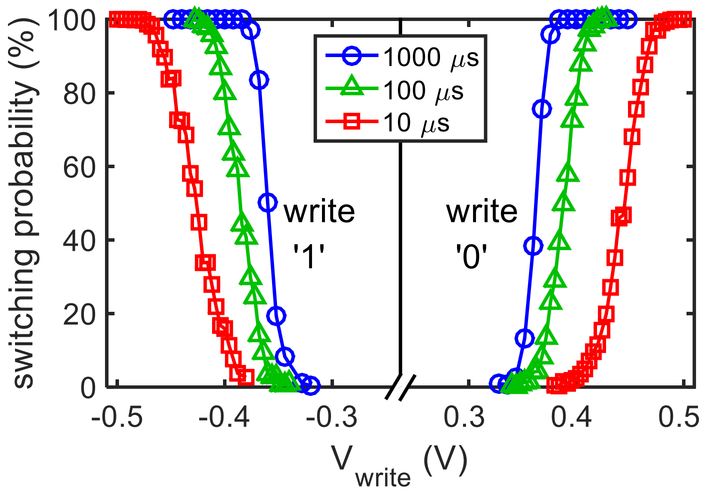

Supplementary Figure S3. Switching probability vs. write pulse amplitude with various pulse widths. Each of the switching probability data points is calculated based on 500 repeats of writing a '0' followed by writing a '1'. Therefore, the effective sample size of the switching probability estimation varies with the switching probability. Generally, a higher switching probability data point bears higher the effective sample size or confidence.

Supplementary Note S4

CRAM logic operation with increased number of inputs. As the number of input cells involved in a CRAM logic operation increases, two mechanisms impact the accuracy. The first is that there are more input states that must be distinguished in the limited range of the relative resistance changes. This makes distinguishing input states more difficult, therefore, impacting accuracy. Note that the input cells are logically capable of producing 2^N states, where N is the number of input cells. However, in terms of the CRAM logic operations, the number of different effective resistance values usually falls into fewer distinguishable groups (assuming all cells have identical electrical properties). For example, three input cells generate eight logic states, but there are only four distinguishable levels of resistance, since there are 0, 1, 2, or 3 cells among all three input cells to be in low or high resistance state. The second mechanism is that, with more input cells, the absolute amplitude of the logic voltage, V_{logic}, required to switch the output cell generally decreases due to the increased number of current paths from the input

cells. Therefore, the portion of V_{logic} on the input cells also decreases. Because the effective TMR ratio of MTJs increases with smaller voltage bias, it leads to an increase in the effective TMR ratio for each input cell compared to a logic operation with fewer input cells.

Supplementary Note S5

CRAM 1-bit full adder design. Figure 4a (in the main text) illustrates the so-called 'MAJ+NOT' design of a 1-bit full adder. The input A, B, and C are first written to MTJ 1, 2, and 3, respectively. This step of loading the inputs is necessary for the experiment but may be optional if the full adder is a subsequent module of some other operations. Then the second stage is a sequence of logic operations. This stage is essential for the full adder. Frist, the majority (MAJ) of MTJ 1, 2, and 3 are produced into MTJ 4. Then the inverse (NOT) of the MTJ 4 is produced into MTJ 5 and 6. Note that this step consists of two steps, and each step is actually a NAND operation between MTJ 4 and 7 into MTJ 5 or 6. The MTJ 7 is loaded with a constant '1' at the beginning of the operations. Lastly, the majority (MAJ) of MTJ 1, 2, 3, 5, and 6 is produced into MTJ 7. At this point, the essence of the full adder is completed, and the results are stored in MTJ 7 and 4, for S and C_{out}, respectively. However, for the experiment study, the results needed to be read out by CRAM memory read operations for further analysis.

The other 1-bit full adder design, so-called 'all-NAND', is shown in Fig. 4b (in the main text). This design is implemented by 9 NAND steps as well as necessary memory write operations to initialize the output cell state before NAND operations.

Supplementary Note S6

CRAM probabilistic model and error simulations. To better understand the impact of error generation, accumulation, and propagation on the full adder designs, numerical simulations were carried out. The simulations are based on the probabilistic models of each logic operation and are implemented by Monte Carlo methods. A probabilistic truth table is used to describe the expected statistical average of the output logical state. For example, a table of [1.0, 0.9, 0.9, 0.1] means that the NAND gate in case of the input being '00', '01', '10', and '11', will on average produce output of 1.0, 0.9, 0.9, and 0.1, or yield accuracy of 100%, 90%, 90%, and 90%, respectively. With the probabilistic truth table (or simply referred to as accuracy) for each logic step defined, more complex operations can be evaluated by simulations. Also note that based on the experimental results, the memory write error and memory disturbance are insignificant compared to the errors associated with logic operations. Therefore, memory operations are assumed ideal in simulations. Then complex Boolean algorithms can be simulated by cascading multiple basic logic operations. And the output results can be statistically analyzed and presented in terms of accuracy. Accuracy can be simply understood as the probability of the output value being as expected.

Figure S4 shows the accuracy of full adder output, Cout and S, vs. input, A, B, and C. When perfect accuracy (100%) is assumed for all logic operations, as shown in Fig. S4a and b, the output accuracy of both full adder designs is also perfectly (100%) accurate. Then finite accuracy of each logic operation step is assumed based on the experimental observations. Specifically, for example, all NAND operations accuracy is set to be the same as the average value of all NAND operations observed in experiments. As shown in Fig. S4c and d, the general pattern of accuracy decay echoes well with the experiment results shown in Fig. 4c and d in main text, that (1) the accuracy of Cout is generally higher than that of S, (2) accuracy for input [ABC] = 000 and 111 is higher than that of the rest of the input states. The simulation results shown in Fig. 4e and f in main text are based on the same simulations described here and with assumptions of accuracy of each logic operation step matching individually to that of experimental observation. To summarize, for MAJ+NOT (all-NAND) design, the simulation results shown in Fig. S4a(b), in Fig. S4c(d), and in Fig. 4e(f) in main text, show that the simulation matches the experimental results increasingly more closely with perfect, uniform finite, and individually matched finite accuracies, respectively.

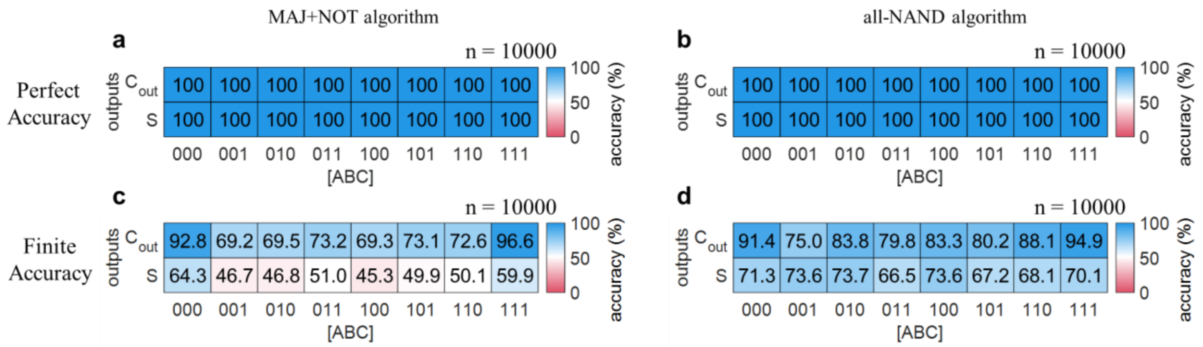

Supplementary Figure S4. Simulated output accuracy of 1-bit full adder operations by CRAM. The CRAM adder's outputs, S and Cout, are assessed against the expected values or the truth table, for all input states of A, B, and C. The accuracy of each result in each input state is shown by the numerical value in black font, as well as, represented by the color of the grid with red (or blue) indicating wrong (or correct), or accuracy of 0% (100%). The accuracy is calculated based on the statistical average of outputs obtained by repeating the full adder execution n times, for n = 10000. A truth table for the 1-bit full adder is listed in black bold font in (**a**) for reference. It is the same for all 1-bit full adder results. **a** and **b** show that if perfect accuracy of each logic operation is considered, the 1-bit full adder accuracy is 100% for all input states. Comparison between **a** (**b**) and **c** (**d**) shows the impact of the 1-bit full adder accuracy when considering finite accuracy for each logic operation.

Supplementary Note S7

Impacts of device-to-device variation on error in a CRAM. There could be several impacts of device-to-device variation. If the input MTJ cells' high or low resistance values are not closely grouped, then the logic states will separate into more levels. This will shrink the margin of accuracy in the worst cases. Another impact of device-to-device variation is disturbances to the data stored in the input cells during a logic operation. Ideally, a logic operation should only alter the state of the output cell while the data in input cells should not be disturbed or destroyed. However, when input cells are not identical, some input cells could carry currents closer to their critical switching current so that they are more likely to be disturbed. For example, if the input and/or output cells happen to be these that are easier and/or harder to switch, the input cells are more likely to be disturbed. And disturbance of data in input cells may result in more errors. Therefore, the reduction or control of device-to-device variation, which can be achieved by utilizing industry-level production processes that are developed and matured for STT-MRAM, is critical and should bring major improvement (reduction) of CRAM accuracy (error rate).

Supplementary Note S8

CRAM logic operation accuracy projections. To project the improvement of CRAM logic operation accuracy due to improvement of TMR ratio, a few models and calculations are involved. Firstly, a basic MTJ resistance model was created to capture the difference in resistance between the states as well as the tunneling behavior found in the AP state. This model is fit to experimental data. The P state resistance is set to the average value measured and is assumed to be constant with respect to the voltage applied across the MTJ. The AP state resistance model is based on the TMR model described in equation 9 of ref. [1]. Assuming constant temperature, fitting parameters C_0 and C_1 are used to fit to the TMR model. The fitted TMR model is combined with R_P to determine R_{AP}. Another fitting parameter, C_2, was added to better match the experimental data. R_{AP} was modeled by fitting Supplementary equation (2) to experimental data using the least squares. Then the C_0 and C_2 are proportionally scaled from the experiment TMR ratio to the target TMR ratio of 200% and 300%.

$$R(V, state) = \begin{cases} R_P, \ state = P \\ R_{AP}(V), \ state = AP \end{cases} \quad (1)$$

$$R_{AP}(V) = \left(\frac{C_0}{100\left(1+\left(\frac{V}{C_1}\right)^2\right)} + 1 \right) * R_P + C_2 \quad (2)$$

The thermal activation model [2,3] is utilized to calculate the switching probability of the MTJ of the output cell under voltage bias. The voltage required for STT to exceed the damping torque is intrinsic critical switching voltage, or V_{C0}. Thermal activated switching occurs when the write pulse amplitude is less than 0.8*V_{C0} and the write pulse width is greater than 10ns. In this regime, thermal fluctuations cause the MTJ to switch with a probability of P_{SW}, which is described by Supplementary equation (3). In this equation, τ is the thermal relaxation time, which is described in Supplementary equation (4). This equation shows that V_P reduces the effective thermal stability factor of the MTJ by a factor of 1-V_P/V_{C0}. In our simulations, P_{SW} was calculated using Supplementary equation (3) and Supplementary equation (4).

$$P_{SW} = 1 - \exp\left(-\frac{t_P}{\tau}\right) \quad (3)$$

$$\tau = \tau_0 \exp\left(\Delta\left[1 - \frac{V_P}{V_{C0}}\right]\right) \quad (4)$$

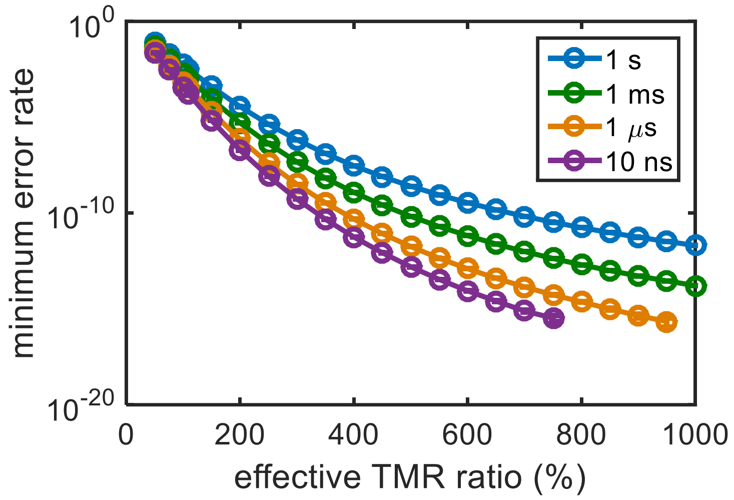

Supplementary Figure S5. Calculated CRAM NAND gate minimum error rate vs. effective TMR ratio with various pulse widths. The 'effective TMR ratio' refers to the perceived TMR ratio under the specific voltage biases each MTJ is subjected to during the NAND operation. This result is obtained with the assumption that the TMR ration is a bias-voltage-independent constant for all MTJs.

Lastly, with the aid of the resistance-voltage model, the current flowing through the MTJ of the output cell during a CRAM logic operation is calculated. This current is then sent to the MTJ switching probability model to calculate the expected average state of the output cell. A sweep for input-state-dependent <D_{out}> vs. V_{logic} is generated and the proper logic voltage and corresponding accuracy for intended logic operation is determined. At a specific V_{logic}, the error rate is minimized. And the corresponding error rate is referred to as 'minimum error rate.' The results for a CRAM NAND gate minimum error rate are shown in Fig. 5a in the main text and Fig. S5. While Fig. 5a in the main text provides a straightforward calculated relationship between minimum error rate and TMR ratio, it is subject to any variations from the TMR model, which may become very different for different material systems and physics. Therefore, the calculation results shown in Fig. S5 assume a voltage-bias-constant

TMR ratio, referred to as ‘effective TMR ratio’, and provide a more material and physics-independent outline for the minimum error rate. It is similar to Fig. 5a that higher TMR ratio and faster logic speed (shorter V_{logic} pulse width) lead to smaller (better) error rate.

Supplementary Note S9

CRAM multi-bit adder, multiplier, and matrix multiplication simulations. Based on the all-NAND full adder, we chain the carry inputs and outputs to establish a ripple-carry adder. A systolic array multiplier using the full adders is also simulated. Using these primitives, a dot-product operation, which is the representative operation for matrix multiplication, is simulated. Figure S6 depicts the dot-product operation with 2-bit fixed-point unsigned inputs, followed by an array multiplier, and a tree adder with conservative bit growth. The adders are chained in a binary tree fashion to minimize the circuit depth. This way, for J inputs, only $\log_2(J)$ levels of adders are needed. We perform the unsigned arithmetic with conservative bit growth, where the output bit size is $2K+\log_2(J)$ for K-bit input elements in a J×J input matrix. We use a certain number of Monte-Carlo trials, where the probabilistic error is injected in NAND gate granularity. In order to compensate for the relatively lower number of trials, we iterate through all input combinations of all circuits in each trial, effectively enabling representative coverage.

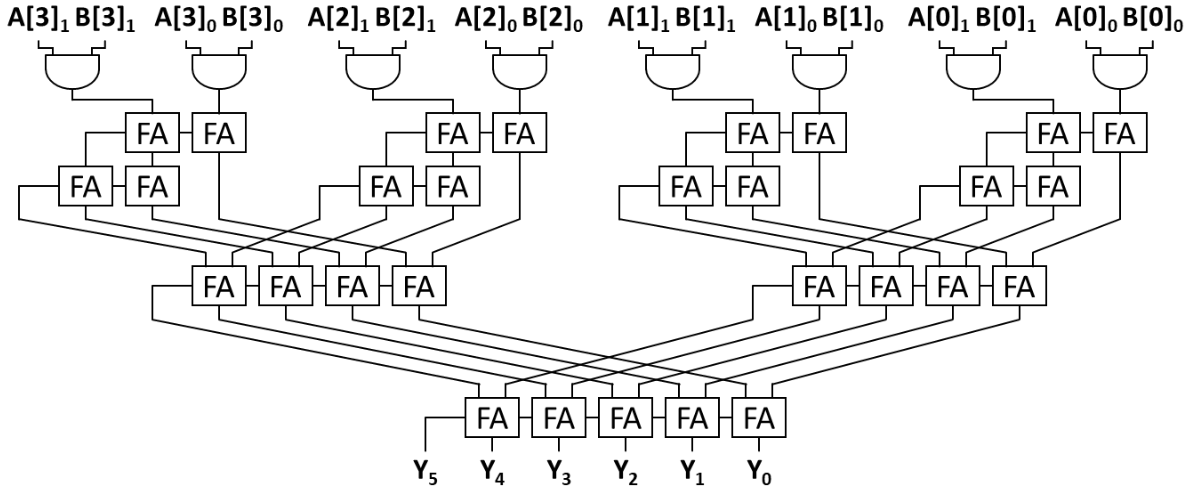

Supplementary Figure S6. Schematics of 2-bit unsigned dot-product computation with 4-element vectors and conservative bit growth.

To evaluate the results, accuracy derived from Normalized Error Distance (NED) metric[4] is used, since it was shown to be more suitable for arithmetic primitives in presence of computational error. The NED accuracy is defined as the NED subtracted from 1. Figure S7 shows the results. The number of Monte-Carlo trials is 1000, 5000, and 1250, for the adder, multiplier, and matric multiplication, respectively. The accuracy generally decreases with increasing input bit width, as they involve more NAND operations. Also, the adder is generally more accurate than multipliers, and matrix multiplications with smaller input sizes are more accurate than these with larger input sizes, for the same reason. Besides the results shown in the main text for 4-bit primitives, at 6-bit, adders yield accuracies of 97.1%, 99.911%, and 99.9968%, for ‘experimental’, ‘production’, and ‘improved’ assumptions, respectively. And multipliers achieve accuracies of 94.2%, 99.80%, and 99.9927%, for the three sets of assumptions, respectively. At 5-bit, the 4×4 matrix multiplications achieve accuracies of 89%, 99.65%, and 99.987%, for the three sets of assumptions, respectively.

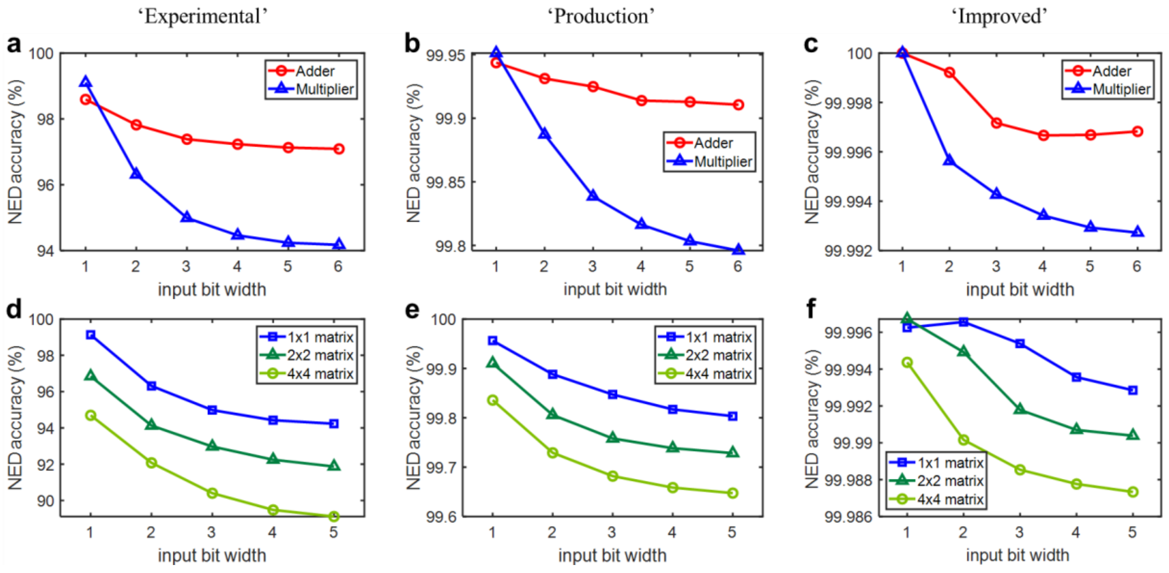

Supplementary Figure S7. CRAM accuracy for multi-bit adder, multiplier, and dot-product matrix multiplication. a, **b**, **c** Normalized error distance accuracy of CRAM adder and multiplier vs. number of bits with assumption sets of ‘experimental’ (a), ‘production’ (b), and ‘improved’ (c). **d**, **e**, **f** Normalized error distance accuracy of CRAM matrix multiplication of various input matrix sizes vs. input bit width with assumption sets of ‘experimental’ (d), ‘production’ (e), and ‘improved’ (f).

Supplementary References

1. Kim, J. *et al.* A technology-agnostic MTJ SPICE model with user-defined dimensions for STT-MRAM scalability studies. in *2015 IEEE Custom Integrated Circuits Conference (CICC)* 1–4 (IEEE, 2015).
2. Diao, Z. *et al.* Spin-transfer torque switching in magnetic tunnel junctions and spin-transfer torque random access memory. *J. Phys. Condens. Matter* **19**, 165209 (2007).
3. Heindl, R., Rippard, W. H., Russek, S. E., Pufall, M. R. & Kos, A. B. Validity of the thermal activation model for spin-transfer torque switching in magnetic tunnel junctions. *J. Appl. Phys.* **109**, 073910 (2011).
4. Liang, J., Han, J. & Lombardi, F. New metrics for the reliability of approximate and probabilistic adders. *IEEE Trans. Comput.* **62**, 1760–1771 (2013).
